# An Optimized Bacteriophage Cocktail Can Effectively Control *Salmonella in vitro* and in *Galleria mellonella*

**DOI:** 10.3389/fmicb.2020.609955

**Published:** 2021-01-21

**Authors:** Janet Y. Nale, Gurinder K. Vinner, Viviana C. Lopez, Anisha M. Thanki, Preeda Phothaworn, Parameth Thiennimitr, Angela Garcia, Manal AbuOun, Muna F. Anjum, Sunee Korbsrisate, Edouard E. Galyov, Danish J. Malik, Martha R. J. Clokie

**Affiliations:** ^1^Department of Genetics and Genome Biology, University of Leicester, Leicester, United Kingdom; ^2^Department of Immunology, Faculty of Medicine Siriraj Hospital, Mahidol University, Bangkok, Thailand; ^3^Department of Microbiology, Faculty of Medicine, Chiang Mai University, Chiang Mai, Thailand; ^4^Department of Bacteriology, Animal and Plant Health Agency, Weybridge, United Kingdom; ^5^Department of Chemical Engineering, Loughborough University, Loughborough, United Kingdom

**Keywords:** *Salmonella*, gastrointestinal enteritis, *Galleria mellonella*, bacteriophage, bacteriophage therapy

## Abstract

*Salmonella* spp. is a leading cause of gastrointestinal enteritis in humans where it is largely contracted via contaminated poultry and pork. Phages can be used to control *Salmonella* infection in the animals, which could break the cycle of infection before the products are accessible for consumption. Here, the potential of 21 myoviruses and a siphovirus to eliminate *Salmonella in vitro* and *in vivo* was examined with the aim of developing a biocontrol strategy to curtail the infection in poultry and swine. Together, the phages targeted the twenty-three poultry and ten swine prevalent *Salmonella* serotype isolates tested. Although individual phages significantly reduced bacterial growth of representative isolates within 6 h post-infection, bacterial regrowth occurred 1 h later, indicating proliferation of resistant strains. To curtail bacteriophage resistance, a novel three-phage cocktail was developed *in vitro*, and further investigated in an optimized *Galleria mellonella* larva *Salmonella* infection model colonized with representative swine, chicken and laboratory strains. For all the strains examined, *G. mellonella* larvae given phages 2 h prior to bacterial exposure (prophylactic regimen) survived and *Salmonella* was undetectable 24 h post-phage treatment and throughout the experimental time (72 h). Administering phages with bacteria (co-infection), or 2 h post-bacterial exposure (remedial regimen) also improved survival (73–100% and 15–88%, respectively), but was less effective than prophylaxis application. These pre-livestock data support the future application of this cocktail for further development to effectively treat *Salmonella* infection in poultry and pigs. Future work will focus on cocktail formulation to ensure stability and incorporation into feeds and used to treat the infection in target animals.

## Introduction

Non-typhoidal *Salmonella* spp. are a leading cause of acute gastroenteritis in humans. Annually, *Salmonella* infection is responsible for ∼155,000 deaths and 93.8 million cases of food poisoning worldwide, of which 85% of all cases are foodborne (Majowicz et al., 2010; Eguale et al., 2015; Balasubramanian et al., 2019). The major route of transmission to humans is via the consumption of food products contaminated with *Salmonella*, especially through poultry and pork related products (Foley et al., 2008). Chickens, turkeys and pigs can become infected with *Salmonella* from contaminated feeds, environment or through contact with other infected animals in the pen (Atterbury et al., 2007). Once the animals are infected, they can remain asymptomatic or develop enteric infection symptoms. Either way, their guts become colonized with *Salmonella* and the bacterium can spread between animals via fecal-oral route (Bonardi, 2017; Martínez-Avilés et al., 2019). In addition to transmission, there is a risk of carcass-to-carcass contamination with *Salmonella* during slaughtering and meat processing (Smith et al., 2018). Consequently, each stage of processing from farm to fork presents a potential risk point of *Salmonella* contamination and infection (Akil and Ahmad, 2019). Thus, breaking the cycle of infection within the food chain before the products are available for consumption represents a desirable approach to prevent and control this infection in humans.

*Salmonella* serotypes commonly associated with poultry and pig infections, and human to human infection via faecal-oral route are *S*. Typhimurium, *S*. 1,13,23:i:, *S*. Enteritidis, *S*. Infantis, *S*. Ohio, and *S*. Seftenberg (Antunes et al., 2016; EFSA, 2018; Ferrari et al., 2019). An increasing number of strains from these serotypes are becoming resistant to the front-line antibiotics used to control *Salmonella* on farms, including to the last resort antibiotic, colistin (Anjum et al., 2016). Worryingly, The European Food Safety Authority (EFSA) reported that 94.4% of 659 *S*. Infantis strains isolated from broilers were resistant to one or more antibiotics, and 64.2% of 123 *S*. Typhimurium strains isolated from pig carcasses were multi-drug resistant (MDR; EFSA, 2018). As a consequence of this, MDR strains have entered the human food chain and alternative antimicrobials are therefore needed to treat and control the spread of MDR *Salmonella* strains in both animals and humans.

Bacteriophages (phages) are natural viruses of bacteria and as such can be developed to offer a viable alternative to antibiotics (Salmond and Fineran, 2015; Czaplewski et al., 2016). Studies have shown that phages are able to lyse MDR *Salmonella* strains (Jung et al., 2017; Thanki et al., 2019; Li et al., 2020) and hence, could be a tool to limit the spread of these strains in the food chain. Due to this increased need for novel antimicrobials, research into the therapeutic use of lytic phages, known as “phage therapy,” has been growing exponentially (Nobrega et al., 2015). To be used most effectively in therapy, phages can be combined as “cocktails” to broaden their host range coverage, improve killing efficiency or limit the development of phage resistance (Chan et al., 2013). Many phage cocktails have been designed against *Salmonella* and their efficacy has been tested in challenge studies both in swine and poultry (Zhang et al., 2015; Martinez et al., 2019). They have been deployed at various intervention points and a pre-slaughter study showed administering a four-phage cocktail in feed (∼10^7^ PFU/g) was able to reduce *Salmonella* colonization in the caecum of chickens by 1 log_10_ CFU/g over 14 days (Sklar and Joerger, 2001). Similarly, a sixteen-phage cocktail (5 × 10^9^ PFU) administered simultaneously with a *S*. Typhimurium (5 × 10^9^ CFU) reduced *Salmonella* colonization by 2–3 log_10_ CFU/g in the caecum, ileum and tonsils of weaning pigs (Wall et al., 2010). In another study, a five-phage cocktail administered post slaughter effectively reduced *S.* Enteritidis on chicken skin by 1.0 log CFU/cm^2^ when administered at the somewhat high multiplicity of infection (MOI) of 10,000 (Hungaro et al., 2013). Finally and rather encouragingly, a similar study showed application of a four-phage cocktail at MOIs of 10 and 100 on pig skin contaminated with *S*. Typhimurium reduced bacterial numbers to below detection levels after 96 h (Hooton et al., 2011).

Although studies have highlighted the use of phage cocktails in reducing *Salmonella* numbers in both pre- and post-slaughter settings, few phage products are available on the market to control infection in poultry and pigs (Żbikowska et al., 2020). For a product to be effective in this setting, it needs to have optimal broad host-range activity to effectively eliminate the diverse *Salmonella* serotypes in animals. Therefore, to address this paucity of information in the control of *Salmonella* infection, here, a novel three-phage cocktail was developed to carry out the first steps needed for the ultimate use of phages as a therapeutic feed additive. The cocktail contains two broad host-range myoviruses and a siphovirus, all of which target prevalent poultry and swine isolates. Clearly, activity of phages *in vitro* may vary *in vivo* and thus further testing in animals is needed. Testing in livestock is expensive and time consuming so to circumvent these difficulties and to reduce the volume of work needed in livestock, the phage cocktail was extensively tested in a *Galleria mellonella* larva *Salmonella* infection model. Previous data from our laboratory and others have shown that the *Galleria* model is useful and that it correlates to large scale animal models. More broadly, the *G. mellonella* model is cheap and is a valuable biological tool to study the virulence and pathogenicity of pathogens including *Salmonella*, and pharmacokinetics of anti-infectives including phage therapy (Thomas et al., 2013; Nale et al., 2016a, 2020). In this study, *G. mellonella* larvae were colonized with representative isolates and detail evidence of the efficacy of different phage therapeutic regimens to prevent and reduce colonization in the model was obtained and is presented.

## Materials and Methods

### Bacterial Strains, and Phage Collation, Isolation and Propagation

In total, 35 *Salmonella* strains were examined in this study. This consisted of twenty-three poultry and ten swine *Salmonella* strains, which were isolated and kindly provided by the Animal and Plant Health Agency (APHA) Weybridge, United Kingdom (Supplementary Tables S1, S2). The phage propagating host, *Salmonella enterica* serovar Typhimurium SL1344 (accession number FQ312003) was obtained from Dr. Primrose Freestone at University of Leicester, and was previously characterized in our laboratory and elsewhere (Viegas et al., 2013; Thanki et al., 2019). *S. enterica* serovar Typhimurium T4, is a routine laboratory strain and was used as a reference strain for phage cocktail development and testing *in vivo*. Bacteria were routinely grown on Xylose Lysine Deoxycholate (XLD) agar (Oxoid, United Kingdom) for 18 h at 37°C before being cultured in Luria-Bertani (LB) broth (Oxoid, United Kingdom) for 18 h at 37°C at 100 rpm.

Twenty-two *Salmonella* phages were tested here. Twenty, were previously isolated and characterized in our laboratory while two, were also previously isolated and characterized in Thailand (Thanki et al., 2019; Phothaworn et al., 2020). All bacterial and phage strains were preserved long term in Viabank cryogenic vials (Abtek Biologicals Ltd., United Kingdom) at −80°C.

### Phage Propagation

To propagate the phages, individual phages were added to separate exponentially growing liquid cultures of SL1344 at OD_600_ ∼0.2 (10^8^ CFU/mL) in LB broth at MOI of 0.1, and further incubated at 37°C with shaking at 100 rpm for 6 h. Cultures of lysed bacterial cells were centrifuged at 5,000 *g* for 15 min, supernatants filtered through 0.2 μm pore size filters (Merch Millipore Ltd. Cork, Ireland) and temporarily stored at 4°C. Phage titers were determined using double agar method with a top bacterial lawn prepared in 4 ml 0.7% LB agar and 150 μL of overnight cultures (produced by inoculating one colony of the bacterial cultures in to 5 mL LB broth and incubated at 37°C for 18–24 h) cast on 1% LB agar 90 mm plates (Kropinski et al., 2009) and expressed as PFU/ml. Equal volumes of phage lysates at the same titers were mixed to form a cocktail.

### Phage Host Range and Virulence Assays on Chicken and Pig Isolates

The host range of each phage was determined by adding 10 μL 10^8^ PFU/mL volumes of lysates to confluently grown bacterial strains prepared as above and incubated aerobically for 18 h at 37°C. Plates were examined for bacterial lysis from three biological and technical replicates.

Five resistant clones obtained from each single phage infection were picked and purified by sub-culturing five times on fresh XLD medium. Each purified clone was confirmed to be resistant if they were no longer susceptible to infection with 10^8^ PFU/mL of the wild-type phage in host range spot testing assay as described above. Confluently grown resistant clones were prepared as above and 10 μL of the wild-type phages were applied to them. Lysis zones were observed after incubation aerobically for 24 h at 37°C.

Phage virulence was determined using killing assay on cultures of SL1344. To do this, 180 μL triplicates of each bacterial culture were produced by diluting 1:10 overnight cultures in sterile LB broth and incubated aerobically with shaking at 100 rpm in a 96-well plate in a SPECTROstar Omega plate reader (BMG LABTECH, Ltd, United Kingdom) set to take readings at 5 min intervals. When OD_600_ ∼0.2 was attained, the cultures were treated with 20 μL of 10^9^ PFU/mL of the individual phage or various permutations of phage combinations (total MOI ∼10). Efficacy of phages to eliminate the bacterial cultures was ascertained by observing lowest reduced growth impacted by treatment of a phage or phage combination. Low OD_600_ readings reveal effective phage killing and this guided the development of appropriate cocktail for downstream virulence as well as *in vivo* assays.

### Optimization of *Salmonella* Infection in *Galleria mellonella* Model

The optimal phage combination developed was tested *in vivo* using the *G. mellonella* larvae *Salmonella* infection model. The larvae were procured, cleaned and prepared as previously described (Nale et al., 2016a). To colonize the larvae with bacterial inocula, cultures of MSG44-S01 (swine), SL1344 (chicken), and T4 (laboratory) strains were prepared in phosphate-buffered-saline (PBS). To do this, a 1:10 dilution of an overnight culture of each strain was prepared in sterile LB broth and incubated aerobically at 37°C until an OD_600_ 0.2 was attained. Cultures were washed three times in PBS by centrifuging at 15,000 *g* for 5 min and resultant pellets re-suspended in PBS each time. The final pellet was resuspended in PBS and diluted to give different bacterial titers and used to colonize *G. mellonella* larvae via oral gavage of 10 μL volumes per larvae using Hamilton pumps as previously described (Nale et al., 2016a).

In order to determine the median lethal dose LD_50_ for each strain in the larva model, a single dose of either, 10^5^, 10^4^, 10^3^, or 10^2^ CFU (in 10 μL volumes) of each bacterial inoculum was administered to duplicate groups of four larvae/per group for each bacterial dose. The infected larvae were incubated at 37°C for 24 h. The impact of bacterial colonization on larval survival was ascertained by scoring for live/dead, and the LD_50_ determined by the concentration of *Salmonella* inoculum required to kill approximately half the number of larval populations in each group within the 24 h time frame. Larvae were considered dead when they become inert and turned black in color (Ramarao et al., 2012; Viegas et al., 2013; Nale et al., 2016a). This dose was selected to initiate colonization for each bacterial strain in the *in vivo* phage therapy studies.

To further optimize the infection model, it was vital to ascertain if the phages were stable within the hemolymphs of the larvae to ensure therapeutic efficacy. Therefore, the stability and survival of the phages within the infection model were determined for the proposed maximum experimental time of 72 h. This time was selected as the larvae will only survive for this long at the stated temperature as previously reported (Nale et al., 2016b). Phage survival within the model was done by treating each larva with ∼10^7^ PFU of the phage cocktail suspension in 10 μL volume using four larvae per 0, 24, 48, and 72 h time points. The treated insects were incubated as described above. Larval survival, and phages numbers within the hemolymph of each larva and from combined feces of larvae from each time point was done using media above and methods previously described (Nale et al., 2016a).

### Phage Therapy Regimens in *G. mellonella Salmonella* Infection Model

Three phage therapy regimens (prophylactic, remedial, and phage/bacterial co-infection), and bacterial- and phage control groups were set up for experimental time points 0, 2, 24, 48, and 72 h for each bacterial strain using four larvae/treatment regimen/time point (Table 1). To initiate colonization, larvae were treated with a 10 μL dose of bacterial inocula used to establish the LD_50_ for each bacterial strain in section “Optimisation of *Salmonella* infection in *Galleria mellonella* model” (10^5^ CFU for SL1344, 10^2^ CFU for MSG44-S01, and 10^3^ CFU for T4) via oral gavage. The three phage therapy regimens were conducted using a single 10 μL dose of the cocktail (at 1:10 bacteria to phage ratio) as previously described for *Clostridium difficile* at time points shown in Table 1 (Nale et al., 2016a). Briefly, in the bacterial control group (Experimental group 1), larvae were treated with the appropriate dose for each bacterial strain at the 0 h time, and at 2 h treated with sterile LB broth. For the phage/bacteria co-infection regimen (Experimental group 2), the larvae were treated with a combination of the phage cocktail and bacteria at the 0 h and followed by LB at 2 h time point. Larvae in the remedial regimen (Experimental treatment group 3) were treated with bacteria at 0 h before being treated with phage at the 2 h time point. For the prophylactic regimen (Experimental treatment 4), larvae were treated initially with the phage cocktail and after 2 h received a bacterial dose. The final regimen is the phage control group (Experimental group 5), here, larvae were treated with the phage cocktail at the 0 h, and at 2 h treated with LB broth (Table 1). After treatments, larvae were incubated at 37°C and remained unfed throughout the experiment (Ramarao et al., 2012; Nale et al., 2016a). At each time points, larvae were scored for survival followed by dissection, and both bacteria and phages were recovered from the hemolymphs on XLD medium using methods previously described (Nale et al., 2016a; Thanki et al., 2019).

**TABLE 1:** Time course of phage treatment regimens on *G. mellonella* used in this study.

**Experimental groups**	**Treatments**	**Time (h)**					
		**0**	**2**	**24**	**36**	**48**	**72**
1	Bacterial control	B	LB	-	-	-	-
2	Phage/bacteria Co-infection	P+B	LB	-	-	-	-
3	Remedial regimen	B	P	-	-	-	-
4	Prophylactic regimen	P	B	-	-	-	-
5	Phage control	P	LB	-	-	-	-

*Four larvae were used for each time point and treatment regimen. Using Hamilton pump, each larva was treated with 10 μL of phage cocktail (P), bacteria (B), co-culture (P + B), or LB broth (LB). Experiment was repeated three times.*

Larval survival, and data for CFU and PFU colonization were analyzed using R and GraphPad Prism version 8 (GraphPad Software Inc, United States). To test efficiency of phage treatment regimens, survival data were analyzed using Log-rank (Mantel-Cox) test. CFU data were subjected to Shapiro–Wilk normality test, and each phage treatment was compared with the bacterial control using Mann–Whitney test. Significance was denoted by asterisks, ^∗^*p* < 0.05, ^∗∗^*p* < 0.01, ^∗∗∗^*p* < 0.001, and ^****^p = 0.0001.

## Results

### Host Range Properties of Examined Phages on Prevalent *Salmonella* Strains Isolated From Chickens and Pigs

The host range of the phages was evaluated to ensure that they provide suitable coverage against a panel of relevant strains and for the proposed use in an agricultural setting. So, they were first used to challenge prevalent strains of which 10 are commonly found in swine and 23 in poultry. Host range was assessed *in vitro* using host range “spot test” on all the strains, and phage killing assays at MOI of 10 on representative isolates as previously described (Hooton et al., 2011). The strains in the panel represent the top five United Kingdom pig and poultry associated serotypes (Supplementary Tables S1, S2).

Among the poultry isolates examined, strains 4–8, 14, and 19–23 were similarly susceptible to all the phages examined as strains were either completely lysed (bacterial strains 5, 7, 8, and 14) or lysed with some resistance on the zones of clearance (strains 4, 6, and 19–23). Although the other bacterial strains 1–3, 9–13, and 16–18 showed variable susceptibilities to the phages, all together the strains were lysed by at least one phage in the collection (Table 2). For the pig isolates examined, strains MSG32-S01, MSG52-S01, MSG29-S01, and MSG41-S01 showed the least susceptibility to infection by the phages as these strains were most resistant or showed partial or cloudy lysis with the phages (Table 3). On the other hand MSG46-S01, MSG57-S01, MSG44-S01, MSG44-S02, and MSG43-S01 showed most susceptibility to the phages with majority of the strain showing either complete lysis or lysis with some resistant colonies observed on zones of clearance as shown in Table 3.

**TABLE 2:** Host range activity of the 22 phages against 23 prevalent poultry isolates examined in this study.

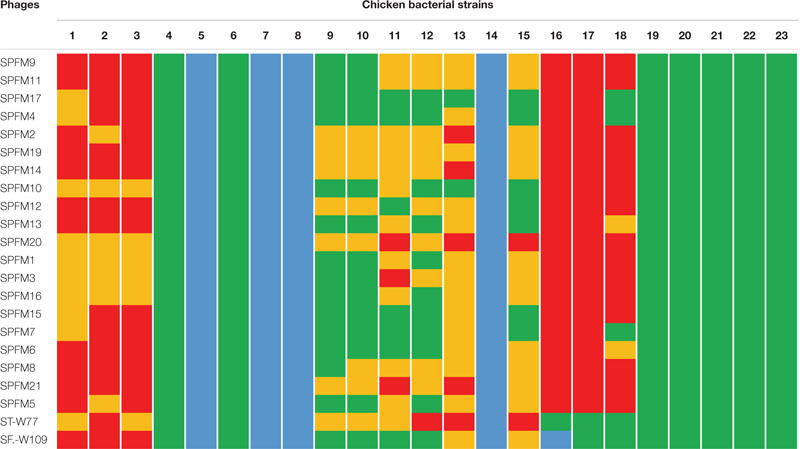

*Confluent bacterial cultures of representative *Salmonella* chicken strains were prepared in 4 mL of 0.7% LB agar medium. Approximately, 10 μL of 10^8^ PFU/mL of phage lysate was applied to the lawn and zones of lysis were observed after incubation at 37°C aerobically for 18–24 h. Key: red = no infection, mustard = cloudy lysis, green = lysis with some resistant colonies observed on zones of clearance, and blue = complete lysis with no resistance.*

**TABLE 3:** Host range activity of the 22 phages on 10 prevalent swine isolates examined in this study.

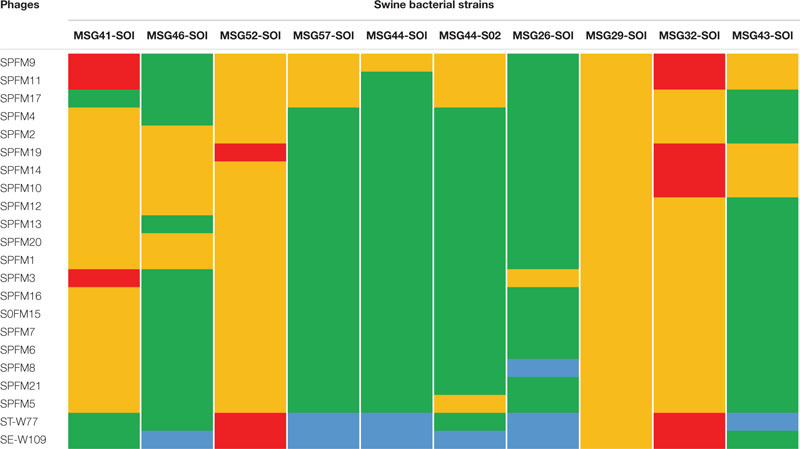

*Confluent bacterial cultures of representative *Salmonella* swine strains were prepared in 4 mL of 0.7% LB agar medium. Approximately, 10 μL of 10^8^ PFU/mL of phage lysate was applied to the lawn and zones of lysis were observed after incubation at 37°C aerobically for 18–24 h. Key: red = no infection, mustard = cloudy lysis, green = lysis with resistance on the zones of clearance, and blue = complete lysis with no resistance.*

Of the phages tested, STW-77 and SEW-109 showed the most efficacy against both pig and chicken isolates lysing (including cloudy lysis, lysis with resistance and complete lysis) 85% (28 out of 33) of the strains tested, which includes multiple serotypes. The remaining phages showed similar lytic activity against most of the strains covering between 60 to 70% of the strains tested (Tables 2, 3).

### Activity of Phages on Growth of *Salmonella in vitro*

To develop a maximally effective phage cocktail, we examined and selected phages with the highest host range activity. Therefore, phages ST-W77 and SE-W109 were selected because of their wide host range activity on the swine and chicken isolates examined (Tables 2, 3). Phage SPFM17 was also included as it was the phage with the widest coverage on the chicken isolates that can also lyse MSG46-S01 and MSG32-S01, which were the swine isolates with the least susceptibility to other phages. Therefore, phage SPFM17 in combination with phages ST-W77 and SE-W109 can lyse over 90% of the bacterial strains from swine and poultry isolates tested (Table 3).

**FIGURE 1:**
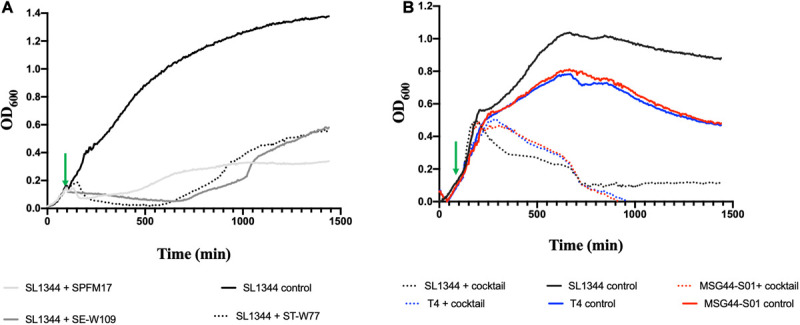
Lysis activity of candidate phages used for cocktail development on bacterial growth of representative swine, poultry and laboratory strains examined in this study. Virulence assay was conducted in 200 μL volumes in SPECTROstar Omega plate reader, containing phage/bacteria at MOI of 10. **(A)** show individual phage killing on SL1344 and **(B)** impact of three-phage cocktail on cultures of chicken SL1344 (black line), swine MGG44-S01 (red line), and laboratory reference T4 strains (blue line). Green arrows represent points when phages were added. Data was analyzed using GraphPad Prism 8.

To examine the complementation effects of the selected phages, all optimizations of cocktail development using the three phages were conducted on the phage propagating host, SL1344, which is also a chicken isolate. When the individual phages were added to the growing culture at OD_600_ 0.2 (at 100 min, indicated with a green arrow), the growth of the bacterium decreased at 100 min after adding phage for all the individual phages and this reduction was maintained for an additional 150 min (for phage ST-W77) and 80 min (for phages SE-W109 and SPFM17) post phage exposure (Figure 1A). However, after ∼200 min (for SPFM17), 500 min (for ST-W77), and 600 min (for SE-W109) post phage treatment, bacterial regrowth was observed (Figure 1A). Next, the individual phage lysates were combined at equal proportions to form a cocktail with the same overall MOI as when phages were used individually, and this was used to infect SL1344 culture at OD_600_ 0.2 at the same growing time of 100 min. The bacterial growth continued to progress for an additional 300 min but then decreased to OD_600_ 0.1 at 550 min post phage treatment (for SL1344) and this level remained consistent until the end of the experiment (Figure 1B). Resistant strains (five clones for each phage treatment) were isolated and challenged with other phages in the mix. It was observed that phage resistant strains produced by one phage was lysed by one or two other candidate phage for the cocktail development (Supplementary Table S3).

Having ascertained the impact of the individual and the three-phage cocktail on cultures of the propagating host, SL1344, we then tested the activity of the cocktail on a swine isolate MSG44-S01, which is fully susceptible to the three phages, and a laboratory strain T4, which is routinely used for most of our *Salmonella* work. The phage cocktail completely eliminated the two additional bacterial strains beyond limit of detection ∼700 min post phage cocktail exposure. This observation remained consistent till the end of the experimental time and no re-growth was observed (Figure 1B).

### Stability of *Salmonella* Phages in *G. mellonella*, and Establishment of Infective Doses of Pig, Chicken and Laboratory Reference Isolates Examined

Having established the efficacy of the cocktail *in vitro* on the representative chicken, swine and on the laboratory test strains, we then tested the lysis activity *in vivo* using the *G. mellonella* larvae. The stability of the phages within the model was ascertained by establishing they were stable and recoverable within the guts and feces of the larvae (Nale et al., 2016a). Our data showed that there was no significant lose in phage titer within the gut of the larvae throughout the 72 h experimental period. Similarly, phages were shed in the feces, albeit a ∼4 log10 PFU/larva reduction was observed 24 h post-exposure but this level remained consistent over the subsequent 48 and 72 time points (Supplementary Figure S1A). Because the bacterial cultures were suspended in PBS before colonizing the larva, we confirmed that the phages were also stable in this buffer as well as no significant loss in titer was observed after resuspending the phages in the buffer for an hour (Supplementary Figure S1B). In addition to determining the phage stability within the larvae and PBS during the *in vivo* model optimization, we further determined the individual strains LD_50_ within 24 h to determine the bacterial numbers needed to cause colonization and to cause death in ∼50% of the larval population within this time frame. This is essential to enable various therapeutic regimens to be tested with the 72 h time frame (Nale et al., 2016a). It was observed that the LD_50_ values were variable for the three strains tested with the lowest being 10^2^ CFU/larva for the swine strain MSG44-S01, which is the swine isolate. However, for T4 and SL1344, 10^3^ and 10^5^/larva, respectively, were required to exert relative LD_50_ effect as in MSG44-S01.

### Impact of Phage Treatment on *G. mellonella* Infected With Various *Salmonella* Isolates

Having fully developed the phage cocktail *in vitro* and optimized the *G. mellonella Salmonella* infection model, the efficacy of the phage cocktail was then tested on larvae colonized with the chicken SL1344, swine MSG44-S01 and laboratory T4 representative *Salmonella* strains. Colonization was established using a single dose of the optimized bacterial culture and followed by various therapeutic phage regimens to determine which treatment would reduce *Salmonella* colonization and enhance survival of the larvae the most.

#### Efficacy of Phage Treatment on *G. mellonella* Infected With Chicken Isolate, SL1344

**FIGURE 2:**
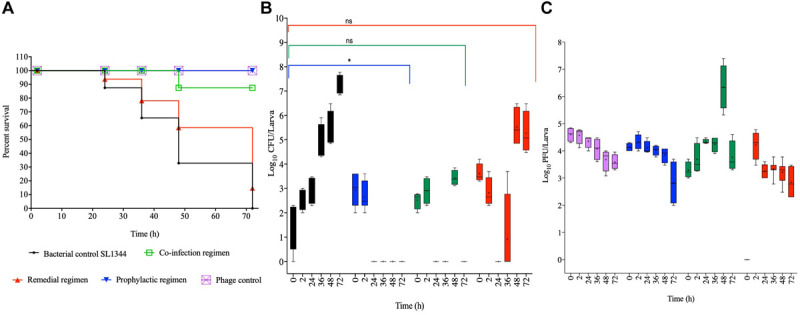
Impact of phage therapy on *G. mellonella* colonized with chicken isolate SL1344. Larvae were colonized with 10^5^ CFU each in 10 μL via oral gavage. Phage therapy regimens were conducted using 10^6^ PFU/larva. **(A)** show survival, **(B)** *Salmonella* colonization, and **(C)** Phage recovery at various time-points for each treatment-Control bacteria (Black), Co-infection (Green), Remedial (Red), Prophylactic (blue) and Control phage (Purple) and Prophylactic (Blue) lines/bars. Four larvae were used for treatment and timepoint. Experiment was repeated thrice and analyzed using Shapiro–Wilk normality test on R. Each phage treatment regimen was tested against SL1344 control using Mann–Whitney test on GraphPad Prism 8. ns = No significance, *significance at *p* < 0.05.

For the SL1344 chicken strain, larval group treated prophylactically survived throughout the experimental time, which is significant compared to the bacterial control group (*p* < 0.0001). Although larval group treated with a co-culture of the phage and bacteria survived until the 36 h, 10% of infected and treated larvae died by the 48th hour but the remaining larvae survived until the end of the experiment. The co-infection regimen is not as efficient as the prophylaxis (*p* < 0.001). The treatment group with the least survival was exhibited by the remedial group, where, ∼95% survived within the first 24 h, and this is not significantly different compared to the control bacterial groups. Continual reduction was observed through the course of the time points, with 86, 60, and 10% survival at the 36th, 48th, and 72nd hour, respectively. The bacterial control group treated with cultures of SL1344 and no phage also showed gradual decrease in survival from 86% at the 24th hour to all larvae dead at the 72nd hour (Figure 2A).

With respect to the colonization of SL1344 within the insects, we observed complete eradication of the bacteria within 24 and 72 h post treatment in the prophylactic and phage bacterial co-culture treatments, respectively. Colonization in the remedial regimen gradually decreased from ∼10^4^ CFU/larva to an undetectable level at the 24th hour, however, bacterial regrowth was observed after 36 h when up to 10^4^ CFU/larva was observed to 10^7^ CFU/larva at the 48 and 72 h times (Figure 2B). When compared with the bacterial control group, prophylaxis regimen was more effective at reducing colonization of this strain (*p* < 0.05) but no significant difference was observed with the co-culture and remedial regimens.

Regarding phage counts, the phage control and prophylactic groups showed a steady level until 48 h followed by a 2 log PFU/larva in the phage control group and a 3 log PFU/larva reductions of phage counts in the prophylactic group at the 72 h time. The phage bacterial co-culture group showed steady phage increase up to ∼10^5^ PFU/larva at the 72 h. In the remedial regimen 10^5^ PFU/larva of phages were recovered at 2 h time but phage numbers later dropped to 10^3^ PFU/larva from the 36th hour to the end of the experimental time of 72 h (Figure 2C).

#### Effect of Phage Treatment on *G. mellonella* Infected With Swine Isolated, MSG44-S01

**FIGURE 3:**
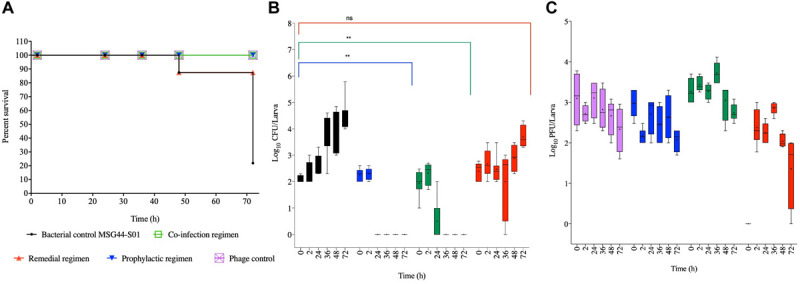
Impact of phage therapy on *G. mellonella* colonized with swine isolate MSG44-S01. Larvae were colonized with 10^2^ CFU each in 10 μL via oral gavage. Phage therapy regimens were conducted using 10^3^ PFU/larva. **(A)** show survival, **(B)** *Salmonella* colonization, and **(C)** Phage recovery at various time-points for each treatment-Control bacteria (Black), Co-infection (Green), Remedial (Red), Prophylactic (blue) and Control phage (Purple) and Prophylactic (Blue) lines/bars. Four larvae were used for treatment and timepoint. Experiment was repeated thrice and analyzed using Shapiro–Wilk normality test on R. Each phage treatment regimen was tested against MSG44-S01 control using Mann–Whitney test on GraphPad Prism 8. ns = No significance, **significance at *p* < 0.01.

For the swine strain MSG44-S01, larvae in the phage control, prophylactic and the co-culture groups all survived throughout the experiment, and both regimens are significant compared to the bacterial control groups (*p* < 0.05). In the remedial regimen and bacterial control, only 13% death at the 48th hour was observed in both groups, which is not significant compared to the bacterial control groups. Although this level remained stable till the 72nd hour time point for the remedial regimen, only 20% larval survival was observed in the bacterial control larval group at this time (Figure 3A).

With the swine bacterial strain, colonization in the bacterial control group progressed from 10^2^ CFU/larva at the beginning of the *in vivo* assay to 10^5^ CFU/larva at the end of the experimental 72nd hour time point. Comparing colonization in larvae within the therapy regimens, it was observed that treating the insect with the phage cocktail prophylactically 2 h before exposing them to the bacteria resulted in the complete prevention of colonization as assessed at the 24th hour. Similarly, administering a phage and bacterial mixture resulted in the eradication of the bacteria at the 36th hour time point, where bacteria were undetectable in the larvae. Consistent with the survival data, both prophylaxis and co-culture regimens significantly eradicated the bacteria from the larvae compared to the bacterial control (*p* < 0.01). In contrast to the other treatments, the remedial regimen was not very effective at eradicating this strain from the larvae as variable colonization levels ranging from undetectable level to 10^5^ CFU/larvae was observed in some of the larvae within this treatment group (Figure 3B). This treatment was not significant compared to the bacterial control group for this strain.

The phage level remained relatively consistent as observed with SL1344 strain, although a 2 log PFU/larva was lost at the 72nd hour in the phage control group, and this pattern is similar in the prophylactic group for this strain. Phage recovery in the phage/bacterial co-infection group increased to 10^4^ PFU/larva at the 36th hour but decreased from the 48th till the 72nd hour time point with 10^3^ PFU/larva recovered. In the remedial regimen with this strain, less phages were recovered and titer decreased from 10^3^ PFU/larva to 10^2^ PFU/larva but increased to 10^3^ PFU/larva at the 36th hour but later dropped to 10^2^ PFU/larvae, however, in other larva phages were not detected in this regimen (Figure 3C).

#### Impact of Phage Treatment on *G. mellonella* Infected With Laboratory Strain, T4

**FIGURE 4:**
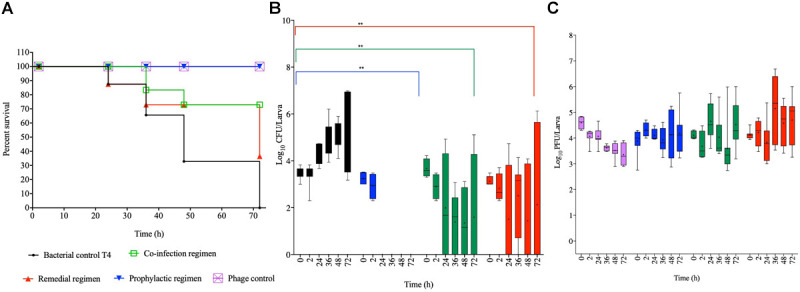
Impact of phage therapy on *G. mellonella* colonized with laboratory isolate T4. Larvae were colonized with 10^3^ CFU each in 10 μL via oral gavage. Phage therapy regimens were conducted using 10^4^ PFU/larva. **(A)** show survival, **(B)** *Salmonella* colonization, and **(C)** Phage recovery at various time-points for each treatment-Control bacteria (Black), Co-infection (Green), Remedial (Red), Prophylactic (blue) and Control phage (Purple) and Prophylactic (Blue) lines/bars. Four larvae were used for treatment and timepoint. Experiment was repeated thrice and analyzed using Shapiro–Wilk normality test on R. Each phage treatment regimen was tested against T4 control using Mann–Whitney test on GraphPad Prism 8. ns = No significance, **significance at *p* < 0.01.

Colonizing the larvae with our reference laboratory strain, T4 and treating with the optimized phage cocktail showed 100% survival among phage and prophylaxis treated larval groups, which is significant compared to the bacterial control group (*p* < 0.0001; Figure 4A). The efficacy of this regimen on this strain is consistent with observations of this treatment regimen in both the chicken and swine isolates shown in Figures 2A, 3A, respectively. With the co-infection regimen, 83% of the larvae survived at 36 h but survival dropped to 72% for the laboratory reference strain at 48 h, and this level remined consistent till the end of the experiment (72 h). This is significant when compared to the bacterial control (*p* < 0.01) but not as efficient as the prophylaxis regimen shown above. The remedial regimen revealed lowest survival with 85% survival at 24 h but this declined to 73% at both 36 h and 48 h, and finally to 36% at 72 h. Although the remedial regimen is the least effective regimen for this strain it is still significant compared to the bacterial control group (*p* < 0.05). The bacterial control showed gradual decline in survival from 87% survival at the 24th hour to complete larval death at the 72nd hour (Figure 4A).

Data on colonization indicated that T4 strain also colonized the larvae with ∼10^3^ CFU/larvae observed at the beginning of the experiment to 10^7^ CFU/larva at the end of 72 h time point. As the pattern in the other two bacterial strains, after 2 h, the bacteria were completely eradicated and were undetected in the larvae in the prophylactic regimen. The co-infection and remedial regimens showed similar colonization levels with starting bacterial count of 10^3–4^ CFU/larva to variable levels at the subsequent time points ranging from undetectable level in some insects to 10^6^ CFU/larva in some at the end of the experiment. All the individual phage therapy regimens significantly eradicated the T4 strain compared to the control (*p* < 0.01; Figure 4B).

Results for phage counts showed higher PFU numbers within the larvae in the remedial treatment group compared to larvae in the other two phage treatment regimens. In all the phage treatment groups, phage counts within the larvae ranged from 10^4–5^ PFU/mL starting phage level to 10^6^ PFU/mL in some of the insects at the end of the experimental time was observed (Figure 4C).

## Discussion

*Salmonella* infection arising from eating contaminated food products remains a major concern to human health with greater percent of cases resulting in mild to severe intestinal gastroenteritis and fatality in others (Sockett and Roberts, 1991; Majowicz et al., 2010). As a result, a number of trade restrictions are introduced in cases where there is contamination with prevalent serotypes of *S.* Enteritidis and *S.* Typhimurium leading to major loss of income to farmers and producers due to rejection of substandard contaminated animal products (Majowicz et al., 2010; Kirk et al., 2015; EFSA Panel on Biological Hazards et al., 2019). Although antibiotics are useful in controlling the infection in both humans and animals, many of the bacterial strains are becoming resistant to routinely used antibiotics leading to treatment failure and disease outbreaks (O’Neil, 2014; Fong et al., 2020). As the identification and development of new antibiotics is slow and difficult, the associated economic and social loss highlight the pressing need to develop alternative more effective therapeutics for this infection (O’Neil, 2014; Romero-Calle et al., 2019). Here, data to support a viable alternative way to control infection in humans is presented. This research focuses on the development of a highly effective *Salmonella* phage cocktail *in vitro* and showing its efficacy in *G. mellonella Salmonella* infection model using various regimens. The data presented here will inform the application of the optimized phage cocktail to effectively prevent or reduce bacterial colonization in animals, thus breaking the cycle of infection and producing safer animal products in the market as previously shown (Wall et al., 2010; Nabil et al., 2018).

The choice of phage therapy approach to control *Salmonella* colonization in animals as proposed in this study has great inherent advantages over conventional antibiotic use. Microbes thrive easily where favorable pH, temperature, moisture and nutrients are present. However, because at ambient conditions or higher temperatures antibiotics efficacy diminishes with time, multiple applications are needed to sustain an effective dose to control a growing bacterial population (Mackowiak et al., 1982; Paterson et al., 2016). In contrast, phages are biological entities, and have been shown to be more stable in various pHs, biotic environments and in ambient conditions than antibiotics (Ahmadi et al., 2017; Sommer et al., 2019). In addition to stability, phages replicate and produce increasing infective particles in the presence of target bacterial pathogen, hence ensuring continuous dosage supply (auto-dosing) of anti-infectives at infection sites (Jończyk et al., 2011; Loc-Carrillo and Abedon, 2011). Furthermore, phages can selectively remove targeted bacteria but exclude other microbial commensals in the niche leaving them unharmed, and this may particularly help animal gut-health, thus producing better quality animal products (Moye et al., 2018; Divya Ganeshan and Hosseinidoust, 2019). Since phages are generally regarded as safe, they are excellent candidates to control *Salmonella* colonization and biofilm development in various ready to eat foods, milk, pigs, and chickens to reduce *Salmonella* colonization (Wall et al., 2010; Huang et al., 2018; Nabil et al., 2018; Islam et al., 2019).

Pertinent to controlling *Salmonella* infection in animals, most previous studies have focused on isolating phages from the environment, testing the activity of individual phages and developing various combinations of phage cocktails with the aim of reducing the bacterial numbers *in vitro* and *in vivo* (Pereira et al., 2016; Islam et al., 2019; Phothaworn et al., 2020). The challenge, however, has been the difficulties of isolating therapeutic phages with acceptable genomic properties, host range coverage and the translation of observed *in vitro* activity to *in vivo* applications in target animals (Nilsson, 2014; Hyman, 2019). All phages examined here are known to be obligately lytic and do not encode undesirable genes expected in a therapeutic phage product (Thanki et al., 2019; Phothaworn et al., 2020). In addition to the genome contents, the phages have been shown to have a wide host range activity on various poultry and swine related *Salmonella* serotypes, thus are excellent candidates for therapeutic purposes in animals (Thanki et al., 2019; Phothaworn et al., 2020). To further ensure that the phages can target the correct strains examined here, they were further challenged with prevalent *Salmonella* serotype strains currently causing infection in pigs and poultry in the United Kingdom, as well as in humans globally (Majowicz et al., 2010; EFSA Panel on Biological Hazards et al., 2019). Despite their variable lysis efficacies on the strains examined in this study, together the phages were able to lyse at least one of the bacterial strains, including the monophasic *S.* Typhimurium strain associated with micro evolution of multi-drug resistance and epidemiologic success (Hugas and Beloeil, 2014; Petrovska et al., 2016; Branchu et al., 2019; Campos et al., 2019; Petsong et al., 2019; Tassinari et al., 2019). These observations concurred with other previous studies which reported phages targeting MDR *Salmonella* strains (Atterbury et al., 2007; Hooton et al., 2011; Jung et al., 2017).

The three-phage cocktail developed here comprised of two myoviruses (SPFM17 and ST-W77) and a siphovirus (SE-W109), indicating that being of diverse morphologies, they may target diverse bacterial host strains resulting to a broad-spectrum cocktail. In synergy to this, our data showed that the individual phages in the mix have complementary contributory target coverage and together lysed 100% of the tested pig isolates, 99.95% of the chicken isolates and combined ∼99.97% of the total serotype strains examined. This suggests that the phages may encode different tail fiber proteins which enabled them to target different receptors on the different host bacteria (Drulis-Kawa et al., 2012). This feature may confer advantage for their therapeutic use as a cocktail, but further work is required to determine this within the genomes of our phage mix. Optimizing our cocktail with diverse phage morphologies concurred with other findings, however, it some cases, single or unknown morphologies were used to construct an effective cocktail (Hooton et al., 2011; Costa et al., 2019; Petsong et al., 2019; Stone et al., 2019). The observed host range coverage of the cocktail spanning various pigs and poultry isolates has been shown in other reports and this further support the prospective multi-purpose application of the cocktail to treat these animals (Petsong et al., 2019). Thus, having both therapeutic and economical advantage to be used in swine and poultry industries.

The phage cocktail we developed has the required host range coverage and has clearly shown efficacy at significantly eliminating the examined bacterial cultures than individual phage treatments *in vitro* using an MOI of 10 (Hooton et al., 2011). For the pig and laboratory reference strains, the phage cocktail completely eliminated the bacterial cultures below the limit of detection, although reduced activity was observed with the chicken isolate. Although enhanced clearance of bacteria using cocktail was reported in previous work on *Salmonella*, other phage cocktails were shown to be no superior to individual phage treatments due to continuous resistance development after treatment in the mix (Hooton et al., 2011; Costa et al., 2019). The observed effective clearance by the optimized cocktail developed here was achieved by a complementation effect, where one phage resistant strain is lysed by another wild-type phage in the mix. This activity concurred with a previous report on *C. difficile*, where resistant/lysogenic strains emanating from one phage infection were efficiently lysed by another phage in the cocktail (Nale et al., 2016b). Although various phage cocktails have been developed for *Salmonella*, this is the first time that this kind of interaction is reported on this species.

The next step in our project was to translate the knowledge obtained on the phage activity *in vitro* into a potential application *in vivo* and to determine which therapeutic regimen would be best in eliminating *Salmonella* in *G. mellonella* model. Therefore, to develop the model for *Salmonella* infection it was essential to begin by optimizing the LD_50_ for each of our test bacterial strain to ensure we have sufficient bacterial load to cause relatively equal effect across the strains tested. Our observation showed that a higher bacterial load of 10^5^ CFU/larva of the chicken strain SL1344 was required to exert comparable LD_50_ effect compared to lower doses of 10^3^ CFU/larva and 10^2^ CFU/larva for the swine and laboratory reference strain. Our observation on the SL1344 chicken isolate concurred with previous *Salmonella* infection work on *G. mellonella* which showed that any dose above 10^5^ CFU/larvae caused death in all larvae within 24 h (Viegas et al., 2013). Except that in our studies we observed ∼50% death in the larvae within this timeframe and this may be attributed to differences in *G. mellonella* type or method of administration. In our study, larvae were colonized via oral gavage while in the previous work colonization was achieved via proleg injection (Viegas et al., 2013).

Comparing treatment regimens, it was clear that prophylaxis was more effective at controlling colonization of all the *Salmonella* strains tested compared to remedial or co-infection with phage and bacteria. This observation is in agreement with *Salmonella* phage treatment in quals and phage therapy studies conducted in the larvae using other pathogens such as *C. difficile* and *Pseudomonas aeruginosa* (Beeton et al., 2015; Ahmadi et al., 2016; Nale et al., 2016a). This observation with *Salmonella* could be attributed to the fact that pre-treating the larvae with the phages for 2 h provided sufficient time for the phage to adapt to the gut environment of the larva as shown in the stability assay, and hence were able to effectively kill the bacteria when administered (Nale et al., 2016a). The other regimens (remedial and co-infection) did not do as well as the prophylaxis and this may be attributed to the ability of *Salmonella* to get intra-cellularized and this may reduce the efficacy of the phages (Diacovich et al., 2017).

## Conclusion and Future Work

Gastro-enteritis caused by *Salmonella* is a major health challenge. The infection is contracted via eating contaminated animal products. Antibiotics are helpful but bacteria are becoming resistant to many front-line antibiotics, hence viable alternative control is urgently needed to reduce the health and economical loss. Here, we reported an approach to the development of an effective therapy using phages to stop infection in animals before products are processed for consumption. To do this we first optimized a broad host-range phage cocktail, which cleared *Salmonella* efficiently *in vitro* and showed that prophylactic treatment regimen is the most effective approach to control the infection in *G*. *mellonella* larva model. The data presented here provides a robust pre-livestock data to support the translation of this cocktail to effectively treat the infection in chickens and pigs. Work is currently ongoing to formulate the phages into pH- and heat-stable powders, and incorporated into feeds and used to control *Salmonella* infection in the target animals.

## Data Availability Statement

The original contributions presented in the study are included in the article/Supplementary Material, further inquiries can be directed to the corresponding author/s.

## Author Contributions

GV and JN conducted the *in vitro* assays. JN and VL conducted the *in vivo* work. AT and PP isolated the phages. MAO, MFA, and AG isolated the chicken and swine isolates. JN, GV, VL, and AT drafted the manuscript. JN and GV analyzed the data. JN, MC, EG, SK, DM, GV, MAO, MFA, AG, and PT conceived and designed the experiments. All authors edited and agreed to be accountable for all aspect of the manuscript and approved the final version to be published.

## Conflict of Interest

The authors declare that the research was conducted in the absence of any commercial or financial relationships that could be construed as a potential conflict of interest.
