# Supplementary material for: An Optimized Bacteriophage Cocktail Can Effectively Control Salmonella in vitro and in Galleria mellonella
Source: Front Microbiol. 2021 Jan 21;11:609955. doi: 10.3389/fmicb.2020.609955 (PMC7858669; doi:10.3389/fmicb.2020.609955)
Supplement: Supplementary file 1 [file Data_Sheet_1.docx]

**Table S1. Swine isolates provided by APHA**

| Strain ID | Serovar | Variant |
| --- | --- | --- |
| MSG41-S01 | 4:i:- | Potential monophasic variant of Typhimurium |
| MSG46-S01 | 4:i:- | Potential monophasic variant of Typhimurium |
| MSG52-S01 | 4:i:- | Potential monophasic variant of Typhimurium |
| MSG57-S01 | 4:i:- | Potential monophasic variant of Typhimurium |
| MSG44-S01 | 4:i:- | Potential monophasic variant of Typhimurium(05-) |
| MSG44-S02 | 4:i:- | Potential monophasic variant of Typhimurium(05-) |
| MSG26-S01 | 4:i:-1,2 | Typhimurium |
| MSG29-S01 | 4:i:-1,2 | Typhimurium |
| MSG32-S01 | 4:i:-1,2 | Typhimurium |
| MSG43-S01 | 4:i:-1,2 | Typhimurium |

Table S2. Poultry isolates porvided by APHA

| **Strain ID** | **Serovar** | **Farm** | **Sample Source** |
| --- | --- | --- | --- |
| 1 | S. 13,23:i:- | Hatchery | Inside macerator |
| 2 | S. 13,23:i:- | Hatchery | Platform - take off area |
| 3 | S. 13,23:i:- | Hatchery | Drain (inside pipe) - take off area |
| 4 | S. Enteritidis | Broiler | House floor |
| 5 | S. Enteritidis | Hatchery | Macerator machine |
| 6 | S. Enteritidis | Layer Hen | Muck - house |
| 7 | S. Enteritidis | Turkey | Dust - house |
| 8 | S. Enteritidis | Turkey | Litter - house |
| 9 | S. Infantis | Broiler | Dust - house |
| 10 | S. Infantis | Broiler | Litter - house |
| 11 | S. Infantis | Broiler | External drain |
| 12 | S. Infantis | Layer Hen | Brushes and spade |
| 13 | S.Ohio | Broiler | Litter – house |
| 14 | S.Ohio | Broiler | Drinkers – house |
| 15 | S.Ohio | Broiler | Drain – external |
| 16 | S. Seftenberg | Hatchery | Hatcher main exhaust |
| 17 | S. Seftenberg | Hatchery | Hatcher discharge gutter |
| 18 | S. Seftenberg | Broiler | Dust – house |
| 19 | S. Typhimurium | Broiler | Litter – house |
| 20 | S. Typhimurium | Turkey | External walkway |
| 21 | S. Typhimurium | Broiler | Bobcat scraper |
| 22 | S. Typhimurium | Broiler | House outlet vents |
| 23 | S. Typhimurium | Broiler | House wall cracks |


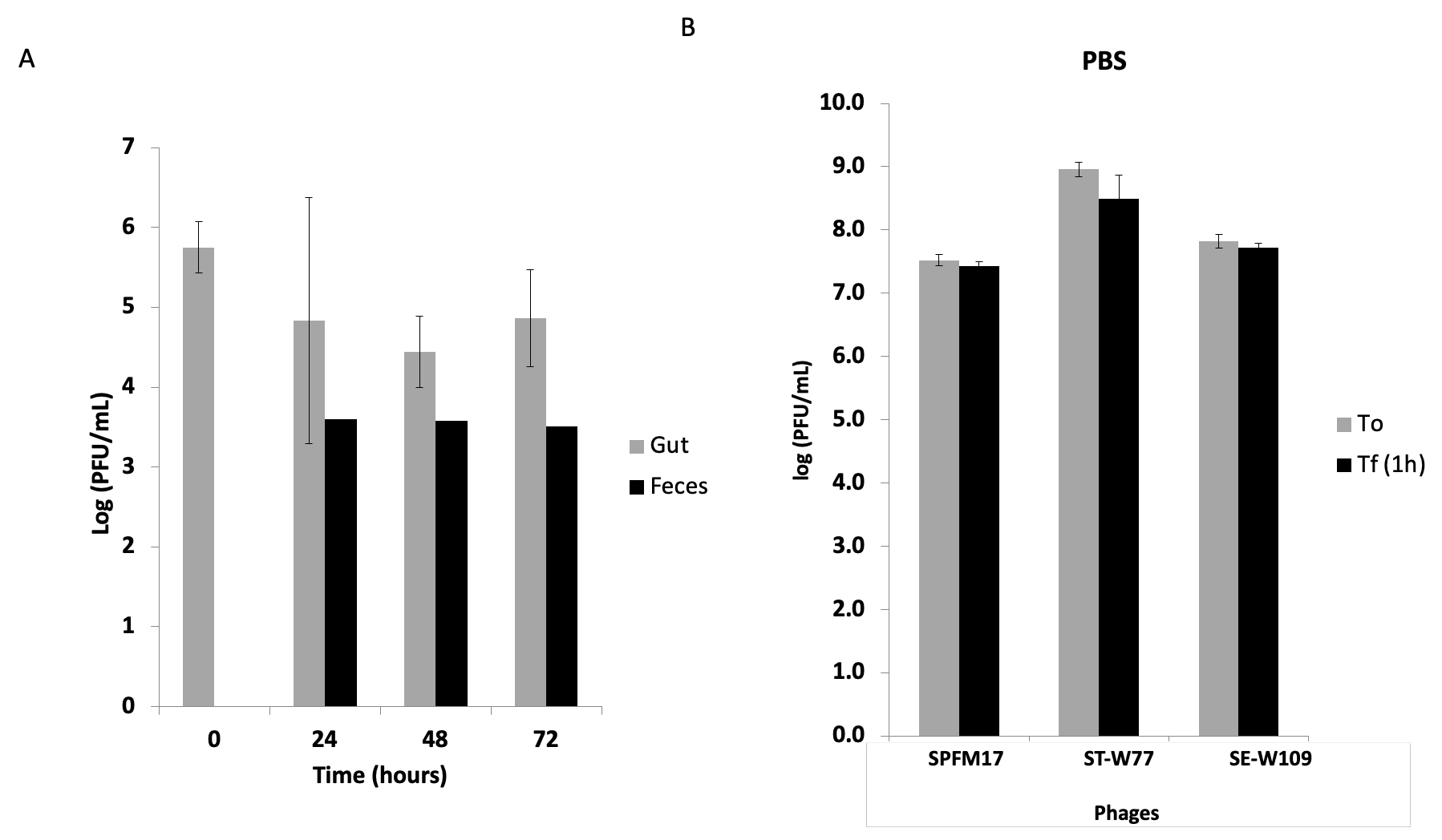


Figure S1 Stability of candidate phages used for cocktail development. A) Stability of phages in the gut of the insect and faces within the experimental time of 72 hours. B) Phage stability in PBS for 24 hours. PBS was the butter used to resuspend the representative *Salmonella* strains before administering to the larvae.

Table S3 Table showing infectivity profiles of phage resistant SL1344 clones by individual phages used in the cocktail development

| **Isolate number** | **Resistant to Phage** | **Activity of wild-type phage** | | | |
| --- | --- | --- | --- | --- | --- |
|  |  | **SPFM17** | **ST-W77** | **SE-W109** | **Cocktail** |
| 1 | SPFM17 |  |  |  |  |
| 2 | SPFM17 |  |  |  |  |
| 3 | SPFM17 |  |  |  |  |
| 4 | SPFM17 |  |  |  |  |
| 5 | SPFM17 |  |  |  |  |
| 6 | ST-W77 |  |  |  |  |
| 7 | ST-W77 |  |  |  |  |
| 8 | ST-W77 |  |  |  |  |
| 9 | ST-W77 |  |  |  |  |
| 10 | ST-W77 |  |  |  |  |
| 11 | SE-W109 |  |  |  |  |
| 12 | SE-W109 |  |  |  |  |
| 13 | SE-W109 |  |  |  |  |
| 14 | SE-W109 |  |  |  |  |
| 15 | SE-W109 |  |  |  |  |

Key

| **Resistant** |
| --- |
| **Susceptible** |
